# Visualizing and Quantifying mRNA Localization at the Invasive Front of 3D Cancer Spheroids

**DOI:** 10.1007/978-1-0716-2887-4_16

**Published:** 2023

**Authors:** Konstadinos Moissoglu, Stephen J. Lockett, Stavroula Mili

**Affiliations:** 1 Laboratory of Cellular and Molecular Biology, Center for Cancer Research, National Cancer Institute, NIH, Bethesda, MD, USA; 2 Optical Microscopy and Analysis Laboratory, Cancer Research Technology Program, Frederick National Laboratory for Cancer Research, Leidos Biomedical Research Inc. for the National Cancer Institute, NIH, Frederick, MD, USA

**Keywords:** RNA localization, Cancer spheroids, 3D invasion, Leader cell, Matrigel, Fluorescence in situ hybridization, Fluorescence microscopy, Single-molecule RNA imaging, RNA quantification, Front-back polarity

## Abstract

Localization of mRNAs at the front of migrating cells is a widely used mechanism that functionally supports efficient cell movement. It is observed in single cells on two-dimensional surfaces, as well as in multicellular three-dimensional (3D) structures and in tissue in vivo. 3D multicellular cultures can reveal how the topology of the extracellular matrix and cell-cell contacts influence subcellular mRNA distributions. Here we describe a method for mRNA imaging in an inducible system of collective cancer cell invasion. MDA-MB-231 cancer cell spheroids are embedded in Matrigel, induced to invade, and processed to image mRNAs with single-molecule sensitivity. An analysis algorithm is used to quantify and compare mRNA distributions at the front of invasive leader cells. The approach can be easily adapted and applied to analyze RNA distributions in additional settings where cells polarize along a linear axis.

## 1 Introduction

Cell migration is a fundamental process that is relevant to a variety of physiological and pathological events. Migrating cells polarize into a leading front, where polymerization of the cytoskeleton drives forward protrusion and a contractile trailing tail that propels the cell forward. Protein components and signaling activities are well known to segregate in a polarized manner between the front and back of migrating cells and to have important roles in orienting and supporting directional motility [1, 2]. In addition, active targeting of mRNAs at the front of moving cells has emerged as a widely used mechanism. Genome-wide screens have identified numerous mRNAs that become enriched in protrusive regions of migrating cultured cells [3–6]. Apart from mRNAs, RNA-binding proteins and components of the translation machinery also become enriched at the protrusive front [3, 7]. Importantly, local mRNA accumulation at protrusions and the localized translation of these transcripts contribute functionally to efficient cell movement [4, 5, 8–11].

mRNA localization at protrusions has been mostly studied in two-dimensional surfaces or modified Boyden chambers with microporous filters [3]. However, cells in vivo typically migrate in three-dimensional spaces where they can engage throughout their surface with other cells or with components of the extracellular matrix (ECM). In these more complex environments, cells encounter various topographies and mechanical properties and, in response, can adopt different modes of migration, which variably depend on signaling mechanisms and cytoskeletal regulation [12–14]. Therefore, study of cellular mechanisms in three-dimensional systems and in assays that recapitulate more complex cellular architectures can reveal principles more pertinent to the physiologic regulation.

Enrichment of specific mRNAs in protrusions of cells has been observed not only on 2D surfaces but also in 3D and in vivo settings, such as during morphogenesis of blood vessels in zebra-fish, in invasive 3D cancer spheroids in vitro, and at the front of invasive tumors in mice [8, 15]. Interestingly, in these latter cases, cells utilize a collective mode of migration, whereby groups of cells move in a concerted manner, remaining connected through cell-cell junctions [16]. Studies in these collective 3D systems have revealed that there is differential regulation of mRNA localization depending on the position and role of individual cells in the context of the whole structure. Specifically, in collectively migrating cellular systems, individual cells adopt different roles by organizing in two functionally distinct groups: leader cells, which are found at the front of invading strands, and follower cells, which remain connected to leaders through mechanical and chemical signaling [16, 17]. mRNAs localized at protrusions, such as the *RAB13* and *NET1* mRNAs, exhibit a prominent localization at the front of the invading leader cell, but not in follower cells. This differential phenotypic behavior cannot be observed when the same cells are examined in 2D culture systems [15].

**Fig. 1:**
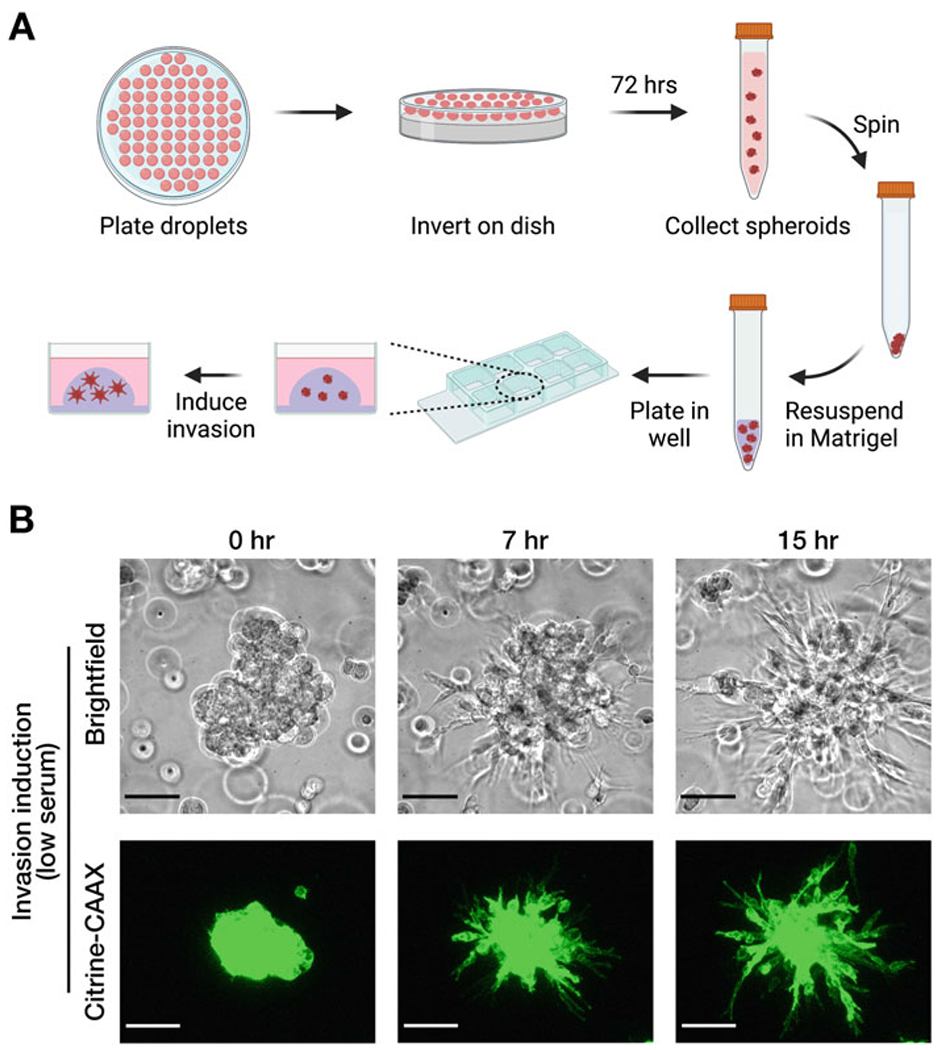
Preparation and imaging of invasive cancer cell spheroids in 3D Matrigel. (**a**) Schematic highlighting the main steps for preparation of spheroids. Groups of cells are plated on the lid of a petri dish, inverted, and incubated as hanging droplets for 72 h. Spheroids are collected, resuspended in Matrigel, and plated as a dome in a chamber slide. For MDA-MB-231 cells, invasion is induced by serum withdrawal. (**b**) Snapshots from time-lapse imaging of MDA-MB-231 spheroids. 0 h. indicates the time of serum withdrawal. Serum withdrawal induces the formation of collective invasion strands within hours. (Upper panels) Spheroids can be visualized by bright-field microscopy. (Bottom panels) MDA-MB-231 cells that express a fluorescent marker (Citrine-CAAX) can also be visualized by fluorescence microscopy. Bottom panel images are maximum intensity z-projections of image stacks through the height of the spheroid obtained on a Leica SP8 confocal microscope. Scale bars: 75 μm

We describe here a method to image and analyze mRNA distributions in 3D cancer spheroids. Spheroid formation relies on the use of the hanging drop culture method, which produces aggregates of cells that can be embedded in ECM (Fig. 1) [18, 19]. As a matrix, we employ Matrigel, a preparation of ECM components extracted from the Engelbreth-Holm-Swarm (EHS) mouse sarcoma [20]. Matrigel components closely resemble the composition of basement membranes since they include laminin-111, collagen IV, entactin, and heparan sulfate proteoglycans [20]. The ability of cancer cells to penetrate and invade into this basement membrane-like environment can recapitulate initial steps occurring during the metastatic dissemination of epithelial tumors away from the primary site. We use MDA-MB-231 breast adenocarcinoma cells, which upon serum withdrawal can be induced to collectively invade into Matrigel in a manner dependent on E-cadherin expression (Fig. 1) [15].

**Fig. 2:**
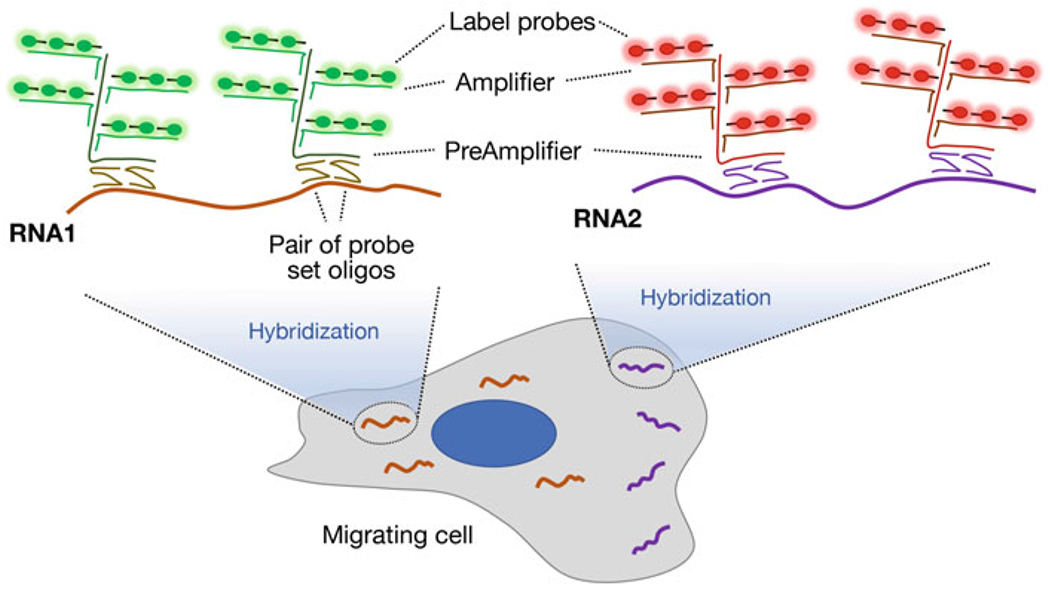
Single-molecule mRNA detection through branched DNA amplification. Target-specific probe sets are synthesized comprising ~20 pairs of oligonucleotides which are designed to hybridize to specific regions within the mRNA of interest (note that only two pairs/mRNA are illustrated). The oligonucleotides of each pair hybridize in adjacent areas so that only hybridization with the correct target mRNA will bring the pair of oligos in proximity, thus enhancing specificity of the subsequent amplification steps. Each oligo pair has regions non-complementary to the target, which when in proximity create a platform that supports the hybridization of a PreAmplifier oligonucleotide. Amplification ensues through hybridization of multiple Amplifier molecules that can subsequently recruit multiple fluorescent Label probes. The resulting branched DNA structure substantially amplifies the signal originating from a single mRNA molecule. Use of PreAmplifier, Amplifier, and Label probes of different sequences, and labeling with distinct fluorophores, allows the simultaneous detection of different mRNA species (RNA1 and RNA2)

Understanding and studying RNA localization requires the ability first to visualize RNA molecules and subsequently to analyze the observed distributions in ways that allow extraction of biologically meaningful information. Various methodologies have been developed to image RNAs with single-molecule resolution [21, 22]. We describe here a method that relies on branched DNA amplification to detect individual mRNAs with robust signal-to-noise ratio [23]. This method is commercialized through Advanced Cell Diagnostics (RNAscope) or ThermoFisher Scientific (ViewRNA^™^ ISH assay). The approach utilizes probe sets of ~20 pairs of oligonucleotides which are designed to hybridize to specific regions within the target mRNA (Fig. 2). Each pair of oligonucleotides hybridizes in adjacent areas and carries additional regions non-complementary to the target. When two probes hybridize next to each other on the target transcript, these additional regions create a platform that supports the hybridization of a PreAmplifier oligonucleotide. This step ensures specificity in target detection and further initiates a series of amplification steps. Specifically, the PreAmplifier contains multiple sites of hybridization with Amplifier molecules that can subsequently recruit multiple fluorescent Label probes (Fig. 2). Thus, through a series of sequential hybridization steps, a branched DNA structure is formed that substantially amplifies the signal originating from a single mRNA molecule. The resulting signal is easily detectable on most available microscope systems.

Available methods to quantitatively describe RNA images rely on identification of individual RNA spots and measurement of various metrics to express their distribution [24, 25]. We present here a method that identifies RNA spots in three-dimensional image stacks and is tailored to quantitatively describe and compare distributions in the context of cells that exhibit front-back polarization [15]. The algorithm computes distances of individual RNAs from specific user-defined front and back boundaries. The distances are normalized to allow comparison between cells and conditions and are statistically compared.

The analysis pipeline presented here is focused on mRNA distributions at the front cytoplasm of invasive MDA-MB-231 leader cells. However, it can be applicable in various other settings of front-back polarization or more broadly in cases of polarization along a linear axis, such as apical to basal. The analysis can additionally be applied in other cell types or methods of RNA imaging.

## 2 Materials

Prepare all solutions using DNase and RNase-free water and molecular biology grade reagents.

### 2.1 Formation of Invasive 3D Spheroids

1. Full culture medium: Leibovitz’s L-15 cell culture medium, 10% FBS, 50 units/mL penicillin, 50 μg/mL streptomycin.
2. Low-serum medium: Leibovitz’s L-15 cell culture medium, 0.1% FBS, 50 units/mL penicillin, 50 μg/mL streptomycin.
3. 37 °C cell culture incubator with atmospheric air.
4. Trypsin-EDTA (0.05%).
5. 1x phosphate-buffered saline (PBS): 155 mM NaCl, 5.6 mM Na_2_HPO_4_, 1.05 mM KH_2_PO_4_.
6. Manual or automatic cell counter.
7. Petri dishes (15 cm diameter).
8. Nunc Lab-Tek II 8-well chamber slides (Thermo Fisher Scientific #154534).
9. Matrigel (Corning #354234).
10. Cell scrapers.
11. Centrifuge for 15 mL tubes.
12. Multichannel pipette and multichannel pipette reservoirs.

### 2.2 Fixation and RNA FISH

1. Fixation solution: 4% paraformaldehyde (EM grade; Electron Microscopy Sciences) and 1x Cultrex cell harvesting buffer (Trevigen #3448–020-01) in 1x PBS (without calcium or magnesium).
2. ViewRNA^™^ ISH Cell Assay Kit components (Thermo Fisher Scientific):
  Detergent solution QC (aqueous buffered solution).
  Probe Set Diluent QF (aqueous solution containing formamide and detergent).
  Amplifier Diluent QF (aqueous solution containing formamide and detergent).
  Label Probe Diluent QF (aqueous solution containing detergent).
  PreAmplifier Mix (DNA in aqueous buffered solution).
  Amplifier Mix (DNA in aqueous buffered solution).
  Label Probe Mix (fluorescent dye-labeled oligonucleotides in aqueous buffered solution).
  Wash Buffer: 0.3% Wash Buffer Component 1 and 0.5% Wash Buffer Component 2 in ddH_2_O.
3. Probe sets for RNA(s) of interest, compatible with ViewRNA^™^ ISH Cell Assay Kit. Probe sets can be purchased from commercially available, existing sets or be custom-designed and synthesized (Thermo Fisher Scientific).
4. Cell mask stain (e.g., HCS CellMask^™^ Green stain; Thermo Fisher Scientific #H32714).
5. ProLong Gold antifade reagent (Thermo Fisher Scientific).
6. Rectangular cover glasses; 50 × 22 mm; No 1.5 (0.16–0.19 mm thickness).
7. Hybridization oven (e.g., UVP HB-1000 Hybridization Incubator).
8. 20x SSC buffer: 3 M NaCl and 0.3 M sodium citrate, e.g., Thermo Fisher Scientific #15557044.

### 2.3 Imaging and Image Analysis

1. Confocal imaging system.
2. Image analysis software (Fiji, MATLAB, RStudio).
3. MATLAB and RStudio analysis scripts (provided as Electronic Supplementary Material).

## 3 Methods

### 3.1 Formation of Invasive 3D Spheroids

#### 3.1.1 Formation of Hanging Droplets

1. Grow MDA-MB-231 cells in full culture medium (*see* Note 1).
2. Detach cells by trypsinization and resuspend in full culture medium.
3. Measure concentration of cells using a manual or automatic cell counter.
4. Dilute cells, using full culture medium, so that there are 25,000 cells/mL. Place cell suspension in a multichannel pipette reservoir or just in a 10 cm plate (*see* Note 2).
5. Using a multichannel pipette, plate 25 μL droplets equally spaced on the underside of the lid of a 15 cm petri dish (Fig. 1a) (*see* Note 3).
6. Within the petri dish, pour 20–25 mL of PBS.
7. Carefully rotate the lid over and place on top of the petri dish, so that droplets are hanging over the PBS (Fig. 1a) (*see* Note 4).
8. Place petri dish into a 37 °C cell culture incubator with atmospheric air (i.e., without injected CO_2_), and incubate for ~72 h.

#### 3.1.2 Embedding Spheroids into Matrigel

1. After the 72 h incubation, prior to starting, thaw Matrigel on ice for 1–2 h. Always keep Matrigel on ice.
2. Place an 8-well chamber slide (or more depending on the number of intended samples) on ice, in a cell culture hood (*see* Note 5).
3. For every sample, pipette 8–10 μL Matrigel into each chamber, and use the tip to evenly spread it around creating a thin layer (*see* Note 6).
4. Place the slides in the incubator for 20–30 min, to allow the Matrigel to solidify.
5. In the meantime, take out the petri dishes with droplets. Turn each lid over and keep it tilted to collect the media and spheroids. Use a cell scraper to sweep droplets toward the bottom, taking care that the liquid does not flow over the edge of the lid. Pool all the liquid and use a P1000 pipette to transfer into a 15 mL tube. Use PBS from the bottom of the dish to wash off and collect remaining spheroids that remain attached on the lid (*see* Note 7).
6. Centrifuge at 100 × *g* for 3 min, to spin down spheroids.
7. Aspirate off medium and place the pelleted spheroids on ice for a couple of min.
8. Add Matrigel (60 μL Matrigel for a spheroid pellet from droplets of one 15 cm lid). Resuspend spheroid pellet in Matrigel by gentle pipetting and by moving the tip around, to minimize breaking up spheroids and to avoid introducing air bubbles.
9. Plate 30 μL of Matrigel/spheroid mix onto the middle of each pre-coated well of the chamber slide, forming a dome-like structure (Fig. 1a). Place in 37 °C incubator for 30 min.
10. Add 600 μL full medium/well to completely cover solidified Matrigel. Return to incubator for 2 h.
11. To induce invasion, remove medium with a pipette, or by careful aspiration, making sure not to disrupt the Matrigel. Wash once with PBS and replace with low-serum medium. Incubate overnight (*see* Note 8).
12. Observe spheroids in a bright-field microscope before and after the overnight incubation to ensure that the spheroid size is appropriate and that invasive strands are apparent (Fig. 1b).

### 3.2 Fixation and RNA FISH

1. After the overnight incubation, prepare a fixation solution and chill on ice. Also, set a hybridization oven to 40 °C.
2. Place the 8-well slide with spheroids on ice and pipette off media.
3. Rinse once with 700 μL ice-cold PBS.
4. Remove PBS and add ice-cold fixation solution (700 μL/well).
5. Keep slide on ice and incubate in a 4 °C cold room for 2 h.
6. Remove fixation solution and check that the Matrigel has dissolved. The dome-like structure should be collapsed and no longer evident (*see* Note 9). Spheroids attached to the glass will appear as bulging areas. All subsequent pipetting steps should be performed slowly to avoid spheroids being washed off (*see* Note 10).
  All subsequent steps are performed at room temperature, unless otherwise indicated.
7. Wash 2x with PBS (all subsequent washing steps are performed using a volume of 0.8–1 mL/well) (*see* Note 11).
8. Add detergent solution QC (200 μL/well) and incubate for 15 min.
9. Wash 2x with PBS.
10. Prepare hybridization solution by diluting appropriate probe sets 1:100 into the Probe Set Diluent QF (pre-warmed at 40 °C). Add 200 μL/well (*see* Note 12).
11. Place slide into a pre-warmed, humidified chamber and incubate at 40 °C for 3 h in a hybridization oven (*see* Note 13).
12. Wash three times, 5 min each, with Wash Buffer.
  If needed, the procedure can be stopped at this stage. After the last wash, replace with 6x SSC and store slides at 4 °C, no longer than overnight.
13. Prepare PreAmplifier Mix solution by diluting PreAmplifier Mix 1:25 in pre-warmed Amplifier Diluent QF. Add 200 μL/well and incubate in a humidified chamber at 40 °C for 30 min.
14. Wash three times, 5 min each, with Wash Buffer.
15. Prepare Amplifier Mix solution by diluting Amplifier Mix 1:25 in pre-warmed Amplifier Diluent QF. Add 200 μL/well and incubate in a humidified chamber at 40 °C for 30 min.
16. Wash three times, 5 min each, with Wash Buffer.
17. Prepare Label Probe Mix solution by diluting Label Probe Mix 1:25 in pre-warmed Label Probe Diluent QF. Add 200 μL/well and incubate in a humidified chamber at 40 °C for 30 min. Protect samples from light for this and all subsequent steps.
18. Wash three times with Wash Buffer, 5 min for the first two times and 15 min for the final time.
19. Prepare a solution of DAPI and cell mask stain in PBS (e.g., 1 μg/mL DAPI; 1/10,000 dilution of a 10 mg/mL HCS CellMask stock solution). Add 200 μL/well and incubate at room temperature for 10 min. Rinse twice with PBS.
20. Remove PBS and use the LabTek slide separator to remove the chambers.
21. Place a small drop of ProLong Gold antifade reagent in each well, and mount using a rectangular coverslip, being careful not to trap air bubbles. Blot excess antifade reagent, if necessary. Let the mounting medium cure at room temperature for 24–48 h before imaging. For longer-term storage, slides can be kept at 4 °C.

### 3.3 Imaging and Image Analysis

**Fig. 3:**
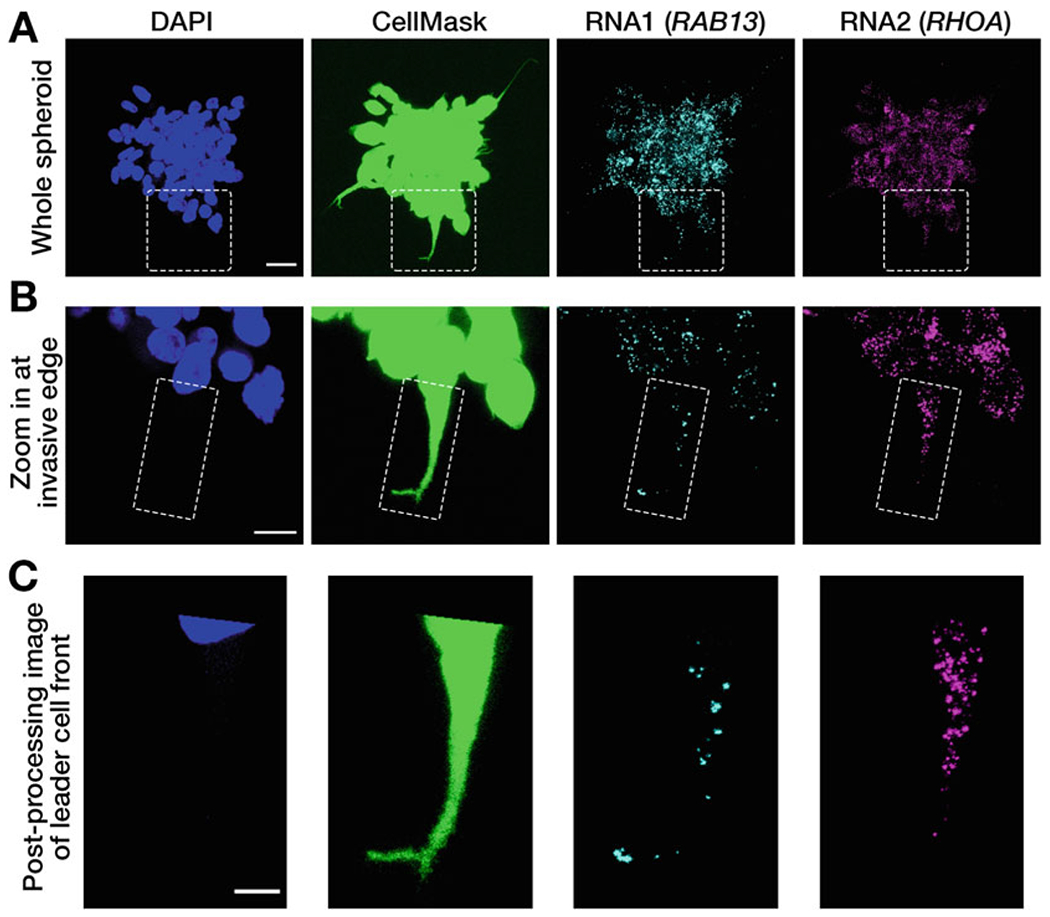
mRNA imaging in invasive cancer cell spheroids and image processing. MDA-MB-231 spheroids were processed by in situ hybridization to detect two mRNA species, RNA1 and RNA2. The presented mRNAs correspond to the *RAB13* and *RHOA* mRNAs. Spheroids were also stained with DAPI to detect nuclei and with CellMask to demarcate the outer, peripheral edges of the spheroid. (**a**) Imaging of the whole spheroid. Confocal image stacks through the body of the spheroid were acquired on a Leica SP8 confocal microscope. Images shown are maximum intensity z-projections. Scale bar 20 μm. (**b**) Zoomed-in images of the invasive area indicated by a dashed box in (**a**). Confocal image stacks through the invasive edge were acquired on a Leica SP8 confocal microscope. Images with well-defined invasive leader cells, such as the one indicated by a dashed rectangle, were further processed for analysis. Images shown are maximum intensity z-projections. Scale bar 10 μm. (**c**) Using the freehand or polygon selection tool in Fiji, an outline was drawn around the front of the leader cell (from the center of the nucleus to the front invasive edge). Outside signal was cleared from all channels and throughout the whole stack, and images were cropped. Scale bar 4 μm. For easier visualization, the images shown are maximum intensity z-projections; however, for analysis the z-stacks are used

**Fig. 4:**
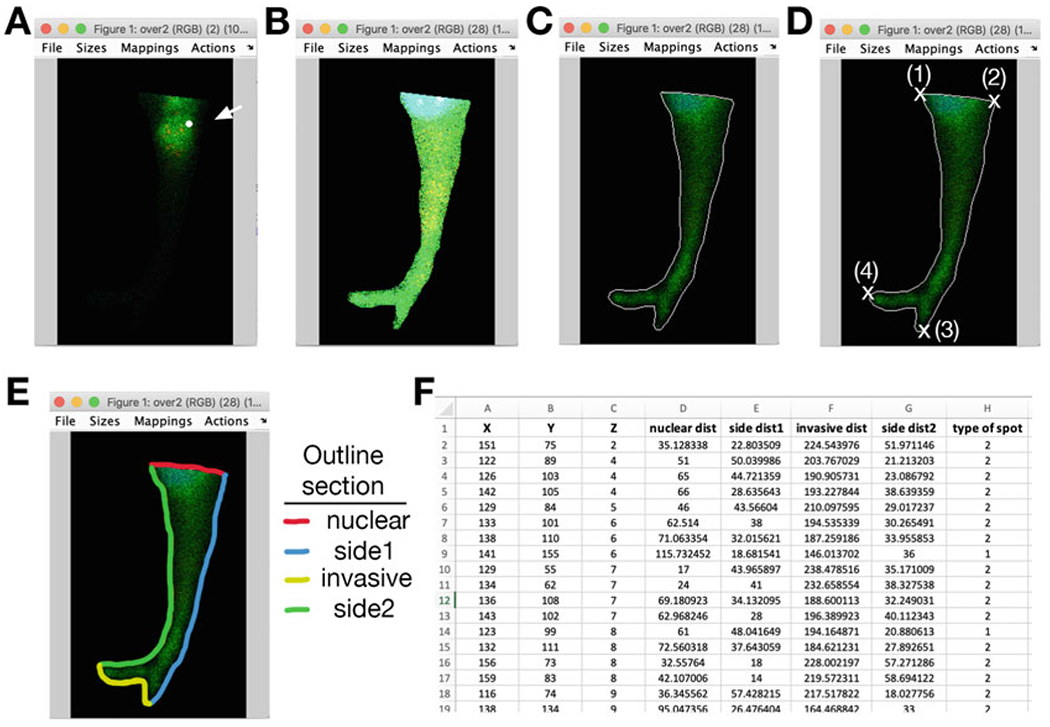
Analysis in MATLAB to measure distances of RNA spots from cell edges. Screenshots of analysis steps are shown. (**a**) Image window displays the image and the first identified RNA spot, whose position is marked by a white dot (arrow). The displayed z-slice is the one where the RNA spot was detected. (**b**) By adjusting the contrast and scrolling through the z-slices, the cell outline can be easily visualized. (**c**) A boundary is drawn to demarcate the cell outline. (**d**) Four points are selected to define the nuclear and invasive sides. It is not necessary to click exactly on the outline (the closest boundary point will be selected by default), but it is important to mark points clockwise starting from the nuclear side, in the order shown. (**e**) The above process segments the outline around the leader cell into four sections: nuclear, side1, invasive, and side2. For each RNA spot, the shortest distance to each of these four boundary sections is computed. (**f**) The coordinates and distances of each RNA spot are saved in a tab-delimited file named “Spot Distances.” An example is shown. The type of RNA spot is also indicated (type 1 for spots detected in channel 3 of the analyzed image; type 2 for spots detected in channel 4)

**Fig. 5:**
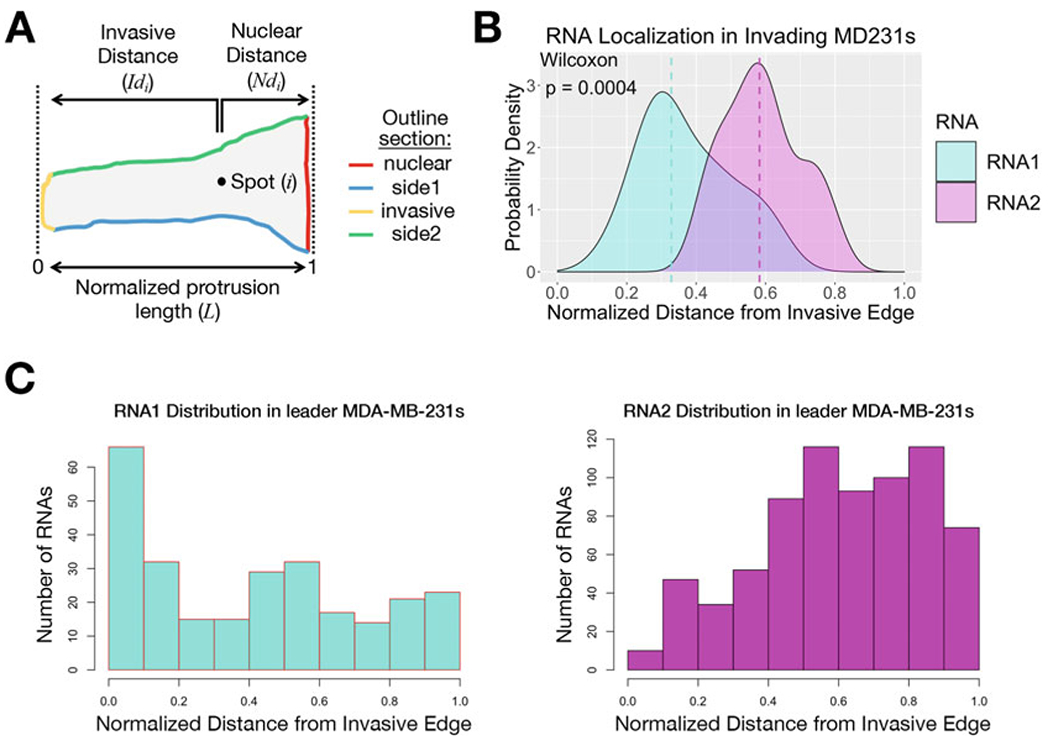
Normalization and analysis of RNA spot distances (**a**) For each image, the total protrusion length is calculated from the invasive and nuclear distances (${Id}_{i}$ and ${Nd}_{i}$, respectively) of individual RNA spots *i*. Distances are normalized such that 0 represents proximity to the invasive edge and 1 proximity to the nuclear edge. (**b**) Plot derived in RStudio. Using values from multiple images, a probability density function that describes the mean normalized distance of each RNA type is plotted, and a Wilcoxon matched-pairs signed rank test statistically compares the distributions. (**c**) Histograms of the normalized distances of all spots included in the analysis

1. Image samples on a confocal imaging system (e.g., Leica SP8) equipped with an appropriate objective (e.g., HC PL APO 63x oil CS2 objective).
2. Acquire four-channel images using appropriate laser lines for excitation and corresponding emission windows (e.g., DAPI channel: 405 nm excitation laser and 420–470 emission; Cell-Mask channel: 488 nm excitation and 495,550 nm emission; RNA1 channel: 561 nm excitation and 570–635 nm emission; RNA2 channel: 647 nm excitation and 655–780 nm emission) (Fig. 3a). The DAPI channel is used to segment nuclei; the CellMask channel is used to define the outline of the leader cell; signal in the RNA1 and RNA2 channels appears as spots which correspond to individual RNAs (*see* Note 14).
3. Identify invasive spheroids and adjust laser power for each channel so that the full dynamic range of the detector is used without saturating any pixels.
4. Acquire sequential z-stacks through the body of the whole spheroid (Fig. 3a). Detectable signal throughout the spheroid body is a good indication that there are no potential issues caused by reduced penetration of the probes.
5. Use digital zoom, or a higher magnification objective, to acquire zoomed-in images of individual invasive cell strands (Fig. 3b). Use a pinhole of 1 Airy unit. It is recommended that the voxel size of the images is around half of the point spread function (PSF) width in the x, y, and z directions. For the system and objective mentioned above, the target voxel size is approximately 0.1 × 0.1 × 0.3 μm (*see* Note 15).
6. Acquire z-stack images covering the whole height of cells in invasive strands (Fig. 3b).
7. Use Fiji/ImageJ software to post-process images for analysis. Using the freehand or polygon selection tool, draw an outline around the front of the leader cell (from the center of the nucleus to the front edge) and clear any outside signal, from all channels and throughout the whole stack (Fig. 3c).
8. Further image analysis in MATLAB requires downloading the DIPimage toolbox (https://diplib.org/DIPimage.html). To install, run dipstart.m (see Electronic Supplementary files) in MATLAB. (Make sure to add DIPimage to the right file path within the Applications folder).
9. Add image file to be analyzed and .m scripts within MATLAB folder (see Electronic Supplementary files; a test image is provided). Information and instructions on running the analysis is provided as an Electronic Supplementary file (see file “info for running 3D spot distance script”).
10. An image window will appear displaying the image and the first identified RNA spot, marked by a white dot (Fig. 4a). The contrast can be altered using the “mappings” menu within the image window, clicking within the window scrolls through the z-slices. Move to a slice that better presents the cell outline (Fig. 4b). A prompt, in the command window, asks if the spot should be analyzed or skipped. To analyze, type “y” and “enter.”
11. Click within the image window and using the mouse draw a boundary to demarcate the cell outline; double-click to end (Fig. 4c).
12. Going clockwise, click to mark two boundary points that define the nuclear side and two boundary points that define the invasive side. It is not necessary to click exactly on the outline (the closest boundary point will be selected by default), but it is important to mark points clockwise starting from the nuclear side (Fig. 4d).
13. All spots within the drawn boundary will be identified and for each spot the nearest distance to each of the four defined sides (nuclear, side1, invasive, side2) will be computed (Fig. 4e) (*see* Note 16).
14. Output values are saved in a tab-delimited file named “Spot Distances” which includes, for each spot, the x, y, and z coordinates, the distance from each side, and the type of spot (type 1 for spots detected in channel 3 of the image; type 2 for spots detected in channel 4) (Fig. 4f). The output files “Spots1” and “Spots2” contain the spots of each individual type together with corresponding intensity values (*see* Note 17).
15. Further processing can be done in RStudio, which can be downloaded at https://www.rstudio.com/products/rstudio/download/. Pool together into a single file the spot values from multiple images, adding a column specifying, with a number, the image that the values are coming from (1, 2, etc.) (see Electronic Supplementary file “Raw-example data.csv”). The file must be a .csv format.
16. In RStudio, run the “Analysis_from_raw_data.R” file (see Electronic Supplementary files). Instructions are included in the script. For each image, the total length (*L*) is calculated as:
$$L=\frac{1}{n}\sum_{i=1}^{n}{Nd}_{i}+{Id}_{i}$$
  where *n* equals the total number of RNAs in a cell and ${Nd}_{i}$ and ${Id}_{i}$ are the nuclear and invasive distance of any individual spot, respectively (Fig. 5a). For each image, a mean normalized distance (*M*) of each RNA type (RNA1 or RNA2) to the invasive edge is determined as:
$$M_{{\mathrm{RNA}}_{1}}=\frac{1}{n}\sum_{i=1}^{n}\frac{{Id}_{i}}{L}\;\;M_{{\mathrm{RNA}}_{2}}=\frac{1}{n}\sum_{i=1}^{n}\frac{{Id}_{i}}{L}$$
  where *n* equals the total number of spots of each respective RNA type in an image. *M* is a number between 0 and 1, with values closer to 0 representing an RNA distribution that is biased toward the invasive edge (Fig. 5a). Across multiple cells, a probability density function (using the kernel density estimation) that describes the mean normalized distance of each RNA type is plotted and saved as a .tif file, and a Wilcoxon matched-pairs signed rank test statistically compares the distributions (Fig. 5b). Using the mean normalized distance of each image for comparisons across multiple cells (instead of pooling the normalized distances of all individual RNA spots) prevents individual cells from having more weight in the results because they include a higher number of detected spots. The script also presents histograms of the normalized distances of all spots included in the analysis (Fig. 5c) (*see* Notes 18 and 19).

## Supplementary Material

TestImage.tif

Raw-example data.csv

localize_spots_in_cell_in_3D_version_3.m

isodata_threshold_3D.m

Info for running 3D spot distance script.docx

find_peaks_above_noise_threshold.m

dipstart.m

Analysis_from_raw_data.R

## 4 Notes

1. MDA-MB-231 cells are used as an example here. Other cell types can be used; however, culture conditions will need to be adjusted accordingly.

2. The concentration of cells at this step will determine the size of spheroids. Adjust accordingly to obtain smaller or larger spheroids.

3. Pipette as many droplets as possible keeping them equally spaced so that subsequent slight movements do not cause individual droplets to merge. The distance determined by the multichannel pipette is a good measure. For a 15 cm petri dish lid, ~4 mL of cell suspension is needed. The material from one such lid will eventually yield two imaging samples. Calculate the necessary cell suspension volume based on the number of needed samples, and make sure to have a slight excess available in the reservoir, for easier and more accurate pipetting.

4. Rotate gently, not too slow, or too fast, so that surface tension keeps the droplets in place.

5. Matrigel solidifies quickly at temperatures >4 °C. While working, Matrigel should be kept on ice, and all surfaces it comes in contact with should be pre-chilled by placing on ice. Pipette tips can be pre-chilled by placing in a 4 °C fridge prior to use.

6. Coating the chamber with a layer of Matrigel prevents spheroids from coming in contact and spreading on the glass bottom. Without it, heavier spheroids sediment before the Matrigel solidifies and contact the glass instead of being fully embedded in Matrigel.

7. Depending on the cell type, the cells within spheroids that are formed in the hanging droplets can be loosely or more tightly adherent. Spheroids with tightly adherent cells can withstand more vigorous pipetting, while loosely adherent ones can become easily dispersed. MDA-MB-231 spheroids are loosely adherent and should be handled gently, minimizing the force and number of pipetting steps.

8. The specific conditions (embedding in undiluted Matrigel and incubation in low-serum media) induce collective invasion of MDA-MB-231 cells. Other cell types invade spontaneously in full media or require embedding in more dilute Matrigel. Optimal conditions must be determined for each cell type employed.

9. Occasionally the Matrigel will not fully dissolve in 2 h. In this case, wash with ice-cold PBS, add ice-cold 1x cell harvesting buffer in 1x PBS and continue incubation at 4 °C overnight. The behavior of spheroids in Matrigel and the ease with which Matrigel dissolves varies based on the specific Matrigel lot. For consistency, it is recommended to initially test different lots of Matrigel and acquire enough of the lot that performs optimally, to carry out a complete set of experiments.

10. Pipette buffers and solutions onto the side of each well letting them flow onto the spheroids. Pipetting directly on the spheroids can lead to detachment.

11. All buffer and solution exchanges should be performed quickly, processing one well at a time. Be careful not to let samples dry as this can lead to increased background noise.

12. Apart from the RNA of interest, it is recommended to detect at the same time a second control RNA, which can be used as an internal reference for quantification.

13. As a humidified chamber, any closed container of appropriate size can be used (e.g., a Western blot box). Place a couple of saturated wet paper towels at the bottom to maintain humidity during incubation. Place in advance the humidified chamber in the hybridization oven, at least 30 min prior to adding the slide.

14. RNA spots are analyzed with the assumption that they correspond to single mRNA molecules. This works well for low-abundance mRNAs, which have around, or less than, 100 copies/cell, such as the examples presented here (Fig. 3).

15. The spatial resolution of a digital image is related to the optical resolution of the microscope (evaluated through the width of the point spread function (PSF)), and the sampling interval (i.e., the number of pixels contained in an image and the distance between them). To preserve the spatial resolution in the digital image, a commonly applied standard suggests that the sampling interval is no greater than half the size of the smallest resolvable feature. For example, for a confocal image acquired using a 63x, 1.4 NA oil objective lens, and an emission wavelength of 570 nm, the width of the PSF in the x and y directions is ~200 nm, and in the z direction is ~700 nm. Therefore, to ensure sufficient sampling, the voxel size would be set to 100 nm in x and y and 300 nm in z. The analysis algorithm uses the assumption of sampling at half the PSF to smooth the image and minimize noise peaks. However, it is expected to perform well even with images that deviate from these values.

16. The algorithm uses the DAPI channel to segment nuclei. Any spots within nuclei are identified and omitted from subsequent calculations, to only focus on mRNA distributions within the cytosol.

17. The number of identified spots can be used as an indicator of how well the default parameters recognize spots in an image of interest. If spots are too few or too many, the noise_threshold_factor values might need to be adjusted (see Electronic Supplementary file “info for running 3D spot distance scripts” for details on the parameters that can be altered). To directly inspect the spots that are being identified, change the “auto” value to 0 so that each identified spot is displayed in the image window, instead of being processed automatically. Clicking “enter” in the command window will sequentially display the slice where a spot is found, and its type, and will present its position in the image window as a white dot.

18. This analysis compares the distribution of two distinct mRNAs within the same cells. To compare the distribution of the same mRNA between different experimental conditions, the values of mean normalized distances (listed in an output file named “Image Means.csv”) can be used for direct comparison and statistical analysis.

19. The analysis described here focuses on leader cells in collectively invading strands. The same analysis can be used to quantify distributions in follower cells. Analysis of follower cells and demarcation of their front and back boundaries can be facilitated by including a staining of a cell surface marker (such as E-cadherin). An immunostaining step with a fluorescently labeled antibody, instead of the CellMask staining step, is recommended.
