# Supplementary figures and images for: Visualizing and Quantifying mRNA Localization at the Invasive Front of 3D Cancer Spheroids
Source: Methods Mol Biol. Author manuscript; Available in PMC 2023 Aug 9. (PMC10411857; doi:10.1007/978-1-0716-2887-4_16)

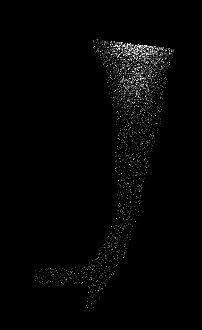

Supplement: TestImage.tif [file NIHMS1919106-supplement-TestImage_tif.tif]
