# Supplementary material for: Visualizing and Quantifying mRNA Localization at the Invasive Front of 3D Cancer Spheroids
Source: Methods Mol Biol. Author manuscript; Available in PMC 2023 Aug 9. (PMC10411857; doi:10.1007/978-1-0716-2887-4_16)
Supplement: Info for running 3D spot distance script.docx [file NIHMS1919106-supplement-Info_for_running_3D_spot_distance_script_docx.docx]

**.m function to localize spots in cell in 3D and measure distance from defined borders**

Stephen Lockett, Leidos Biomedical Research (c) 2017

For research use only

Run under MATLAB 7.6.0.324 (R2008a) (requires license) and DIPimage 2.7 or later

Required MATLAB toolboxes: NONE

**Assumptions:**

(1) Image is 4D, x, y z and color

(2) Image is ordered as follows: channel 1: nuclei, channel 2: cell mask, channel 3: RNA1, channel 4: RNA2.

(3) Voxel size is half the PSF width in the x, y, z dimensions

To run, paste the following in MATLAB’s command window:

(You need to define the name of the image file to be analyzed (make sure the image file is in MATLAB’s folder). You might have to adjust the noise threshold factor values. Also, the auto value can be set to a value higher than the number of your z-stack slices, so that you draw the border only once and automatically calculate all spots. See next page for additional details.)

name_of_image_file = 'TestImage.tif'

noise_threshold_factor_1 = [10,400,800];

noise_threshold_factor_2 = [10,400,800];

spots_spreadsheet_1 = 'spots1'

spots_spreadsheet_2 = 'spots2'

type_of_spots = 1;

find_peaks_above_noise_threshold(name_of_image_file, noise_threshold_factor_1,type_of_spots, spots_spreadsheet_1)

type_of_spots = 2;

find_peaks_above_noise_threshold(name_of_image_file, noise_threshold_factor_2,type_of_spots,spots_spreadsheet_2)

name_of_output_image = 'temp3'

isodata_threshold_3D(name_of_image_file, name_of_output_image)

name_of_output_spreadsheet = 'SpotDistances'

number_of_spots_to_skip = 0;

auto = 200;

localize_spots_in_cell_in_3D_version_3(name_of_image_file, spots_spreadsheet_1, spots_spreadsheet_2, name_of_output_image, noise_threshold_factor_1, noise_threshold_factor_2, name_of_output_spreadsheet, number_of_spots_to_skip, auto)

**INPUTS AS FUNCTION ARGUMENTS**

(1) name_of_image_file: Name of 4D image stored as a stacked tif file.

Color order must be: nuclei, cell mask, RNA1, RNA2

(2) spots_spreadsheet_1: Excel spreadsheet containing the (x,y,z)

coordinates of the RNA1 FISH signals. (Generated by the MATLAB function find_peaks_above_noise_threshold)

(3) spots_spreadsheet_2: Excel spreadsheet containing the (x,y,z)

coordinates of the RNA2 FISH signals. (Generated by the MATLAB function find_peaks_above_noise_threshold)

(4) segmented_nuclei: MATLAB .mat file containing the segmented nuclei. (Generated

by the MATLAB function find_peaks_above_noise_threshold)

(5) noise_threshold_factor_1: Three element variable for segmenting the RNA1 FISH signals.

(i) Ratio of intensity of peak to surrounding background (recommend 10), (ii) minimum allowed peak intensity and (iii) always a peak if above maximum.

For example: for noise_threshold_factor [a,b,c]:

b: if intensity of RNA spot<b it is not considered; if intensity >c it is always considered; intensities between b and c are considered only if the peak intensity of the RNA spot is >a times the average intensity of the surrounding background

(6) noise_threshold_factor_2: Three element variable for segmenting the RNA2 FISH signals, as above

(7) name of output spreadsheet (‘SpotDistances’ by default)

(8) Number of spots to skip (use only if image stack contains spots outside of the cell of

interest in the initial z planes, otherwise leave to 0)

(9) auto: automatically localize spots in pre-existing drawn regions.

0: manual (always draw region for every spot); 1: automatic using only spots detected in

the same slice; 3: automatic using spots centered in the same slice or

one above or one below; 5: 2 above or 2 below; etc.

(This can be useful if the shape of the cell differs significantly between z-slices and it is

desired to define different outlines at different z-positions. In most cases, drawing only

one outline is sufficient since the front of invasive leader cells is not very wide. In this

case, insert a number equal or larger than the slices included in the analyzed z-stack

(the default is 200), so that the outline is drawn only once)

**OUTPUT**

Tab and comma delimited spreadsheet of the tabulated x, y, z coordinates of each analyzed

spot, distances to the borders (spot to nucleus, spot to side1, spot to invasive front, spot to side2) and type of spot: 1 = RNA1 (channel 3), 2 = RNA2 (channel 4)
